# IgG4-mediated sclerosing fibroinflammatory disease presenting as inflammatory breast malignancy

**DOI:** 10.1259/bjrcr.20180041

**Published:** 2019-04-16

**Authors:** Muram El-Nayir, Ash Subramanian, Zainab Ali, David Howlett

**Affiliations:** 1 East Sussex Healthcare NHS Trust, Eastbourne, UK

## Abstract

IgG4-mediated sclerosing fibroinflammatory disease is a rare systemic disease which has the ability to form masses in multiple organs and may mimic malignancy. In this case we describe a 53-year-old female who presented with clinical and imaging findings in her right breast consistent with inflammatory breast carcinoma and associated right axillary nodal mass. She underwent CT which also uncovered a left thyroid mass and suggested both masses were possibly malignancies. She proceeded to ultrasound-guided core biopsy of each, which showed an appearance characteristic of IgG4-mediated sclerosing fibroinflammatory disease. The patient was treated with steroids with good outcome. This is the first described case of this condition presenting in this way to our knowledge and this diagnosis should be considered in patients with similar presentations.

## Introduction

IgG4-mediated disease is a rare, autoimmune, fibroinflammatory process and essentially an umbrella of diseases with numerous different manifestations. It may involve multiple organs with the formation of inflammatory masses which may be initially misinterpreted. This case is an unusual manifestation of this uncommon disease.

## Case report

A 53-year-old female presented with increasing swelling and pain of her right breast over several months. She otherwise had no other symptoms with no significant past medical history.

On examination, her right breast was enlarged, swollen and tender over the upper outer quadrant, with erythematous skin thickening and changes consistent with peau d’orange. There was no nipple change or arm lymphoedema. She was found to have a large mass in the right axilla (5 × 6 cm), which was hard and fixed on palpation suggestive of pathological lymphadenopathy. A clinical diagnosis was made of likely inflammatory breast carcinoma, with metastatic axillary lymph node involvement. No other clinical findings were identified.

Her initial blood tests were normal including full blood count, thyroid-stimulating hormone, follicle-stimulating hormone, luteinising hormonel, testosterone and 17 Beta oestradiol. In particular, she had no peripheral eosinophilia with a normal eosinophil count and normal inflammatory markers.

**Figure 1.**
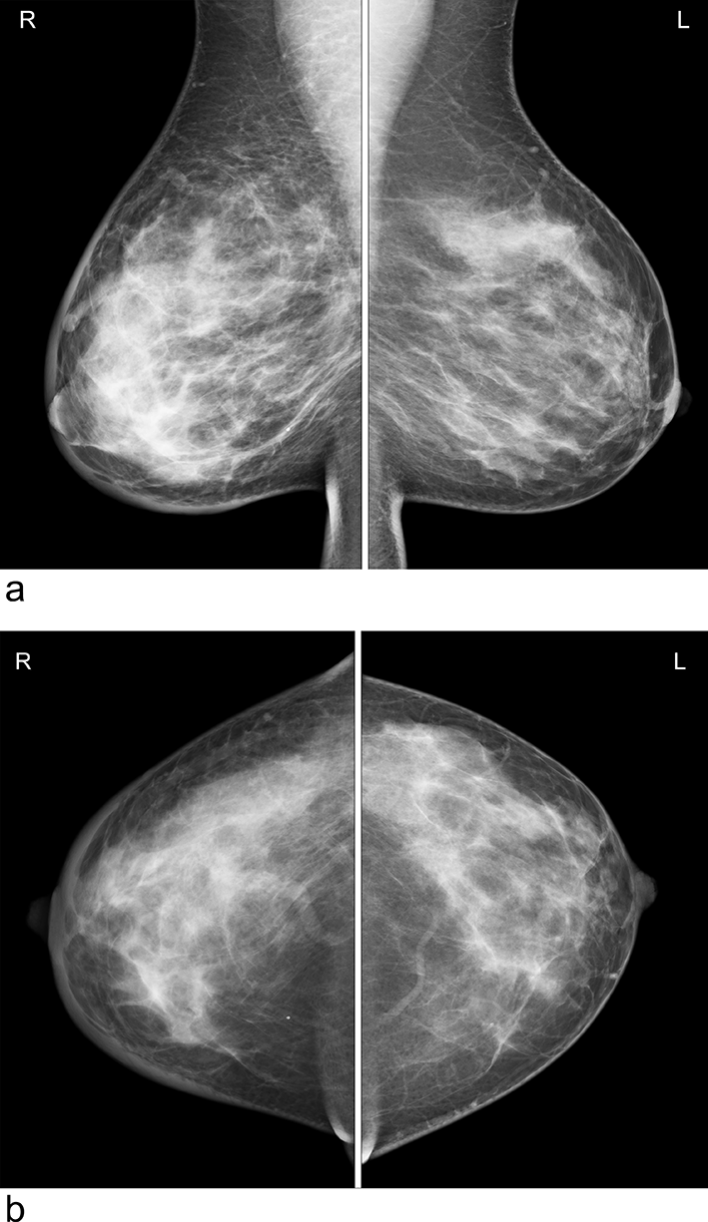
Lateral oblique and craniocaudal mammograms. Note abnormality in right breast texture, skin thickening and trabecular coarsening and distortion. No definite mass. Left breast appears normal.

**Figure 2.**
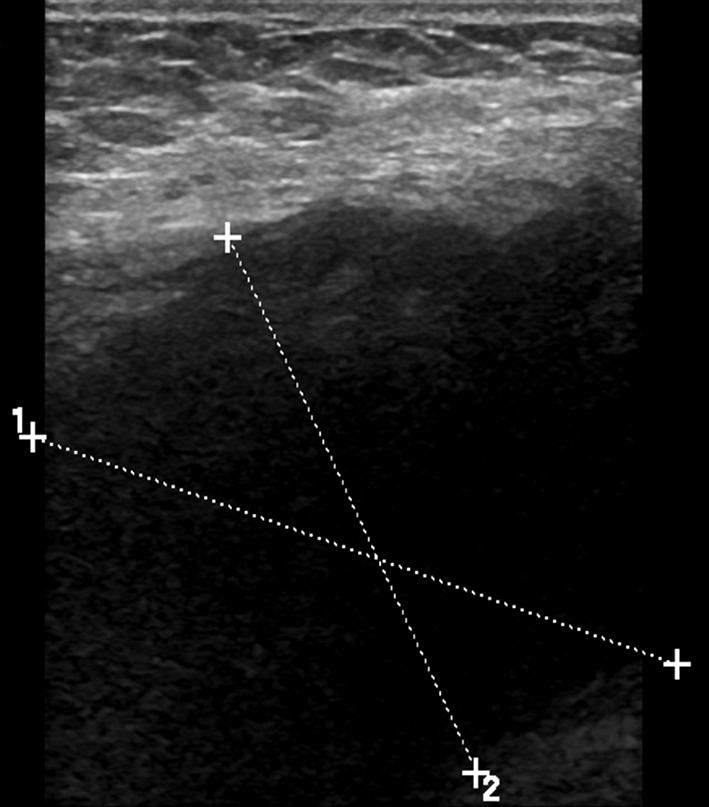
Ultrasound right axilla shows a poorly defined hypoechoic mass (callipers).

In accordance with clinical findings, the patient underwent breast imaging. Mammography demonstrated oedema type changes in the right breast with trabecular coarsening and skin thickening, but no discrete masses were identified (Figure 1a,b). Ultrasound of the right breast confirmed generalised oedematous changes and skin thickening, but no focal mass was identified. Ultrasound of the right axilla confirmed a large vascularised hypoechoic mass suggestive of malignant lymphadenopathy (Figure 2). She proceeded to ultrasound-guided core biopsy of the axillary mass (14G) and also underwent CT of the neck, chest, abdomen and pelvis to stage her presumed breast tumour.

**Figure 3.**
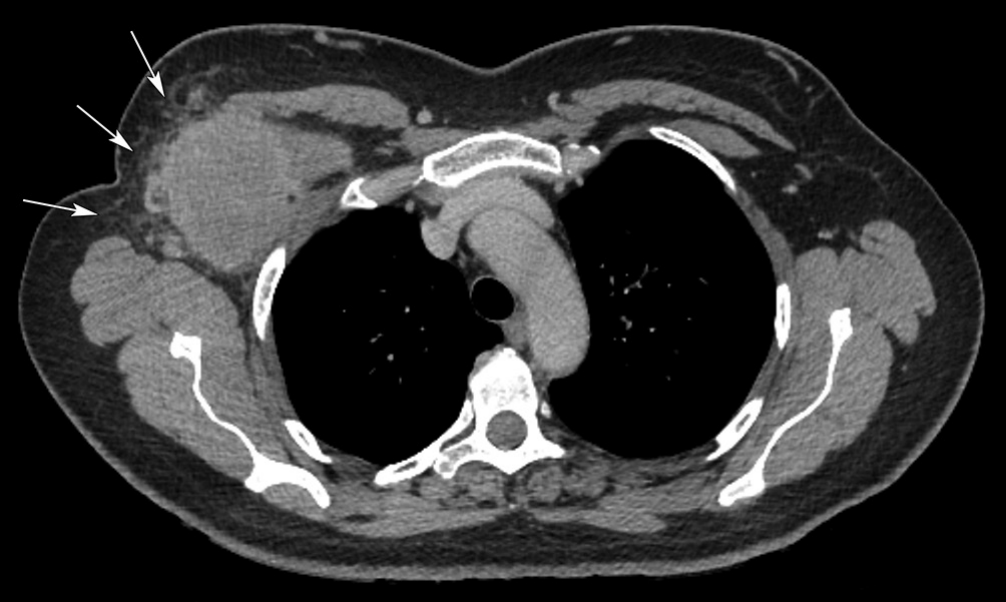
Axial post contrast CT of the axillae, confirms a poorly defined soft tissue mass in the right axilla. Note distortion and stranding of the overlying subcutaneous fat (arrows) and infiltration of chest wall/pectoralis minor musculature.

**Figure 4.**
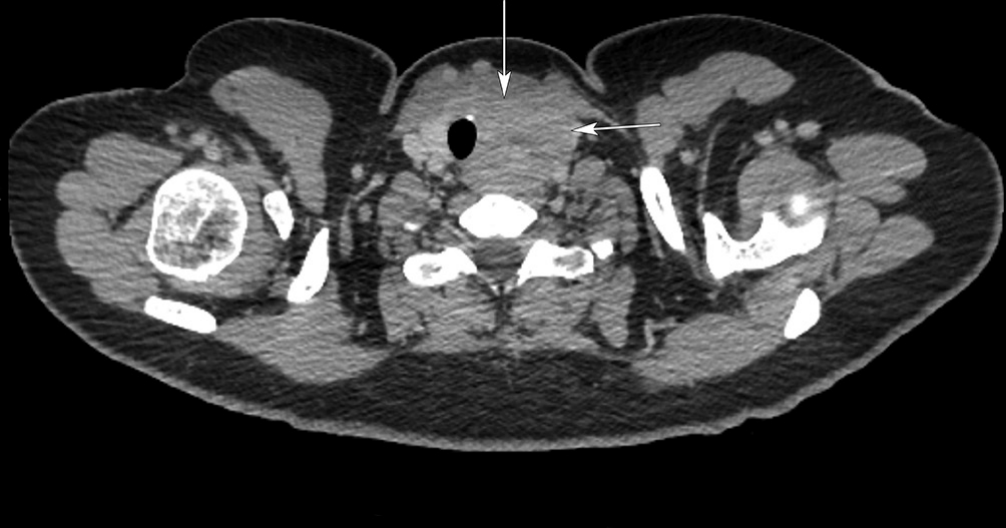
Axial post contrast CT thyroid, shows a poorly defined soft tissue mass in the left thyroid (arrows).

**Figure 5.**
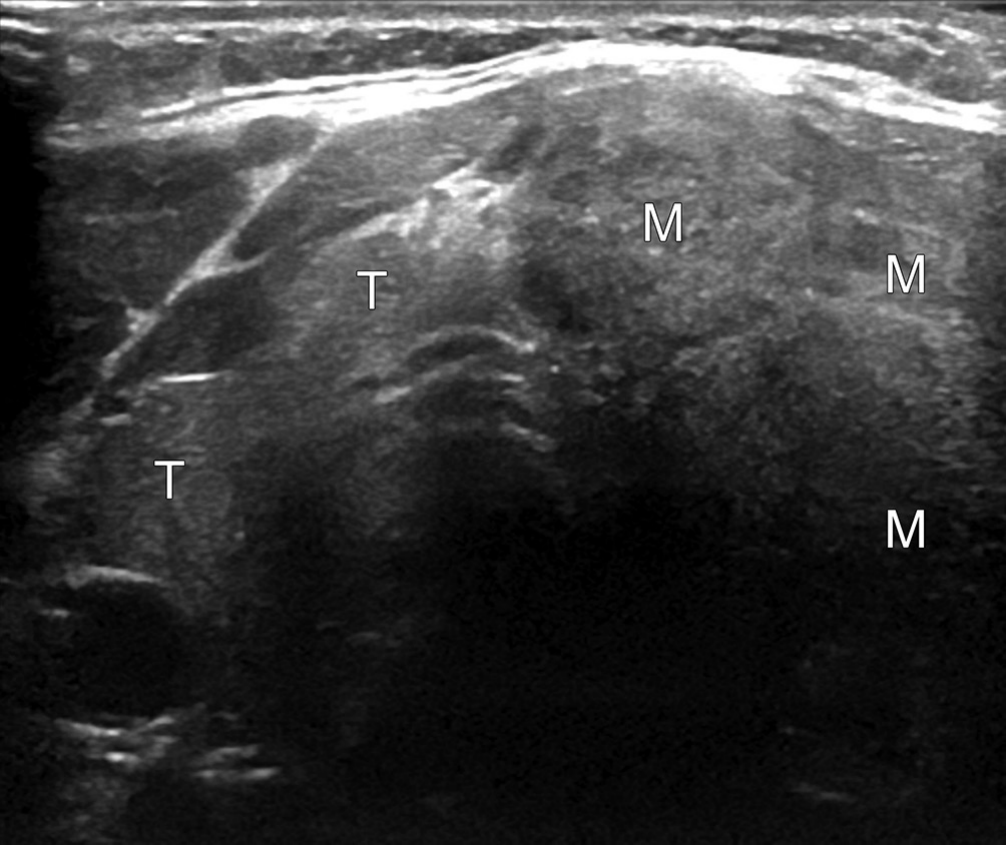
Transverse sonogram of the thyroid. Normal right lobe and isthmus (T). There is a large mixed echogenicity mass partly delineated in the thyroid left lobe (M).

CT confirmed a malignant looking mass in the right axilla with no evidence of distant metastasis (Figure 3). It also demonstrated an ill-defined soft tissue mass in the left thyroid (Figure 4). The possibility of thyroid malignancy was considered and the patient also underwent ultrasound and ultrasound-guided core biopsy of the thyroid lesion (Figure 5).

**Figure 6.**
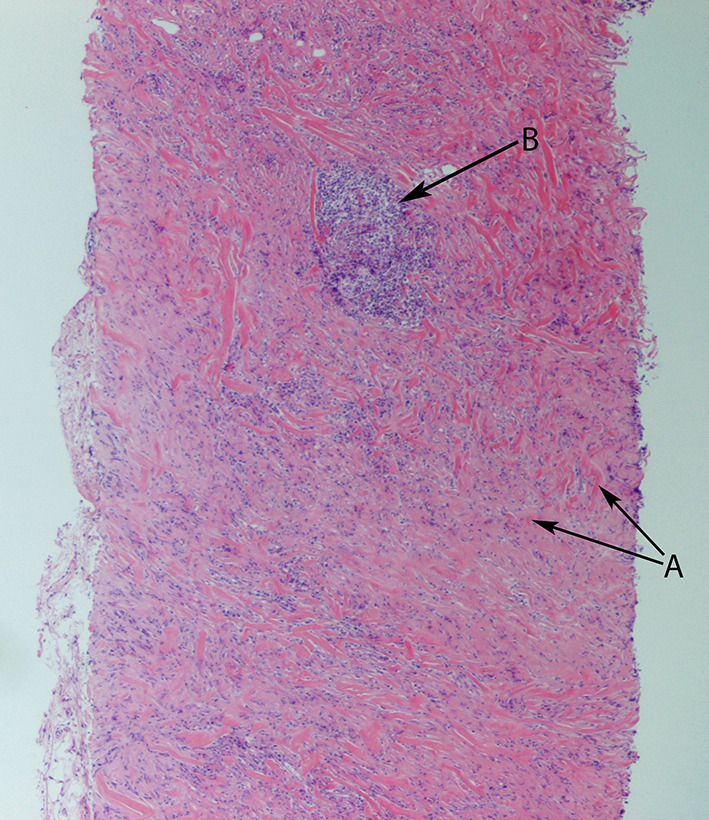
Thyroid gland core biopsy specimen shows dense fibrosis with interspersed chronic inflammatory cells. A: fibrosis. B: chronic inflammation (H&E ×10). H&E, haematoxylin and eosin stain.

**Figure 7.**
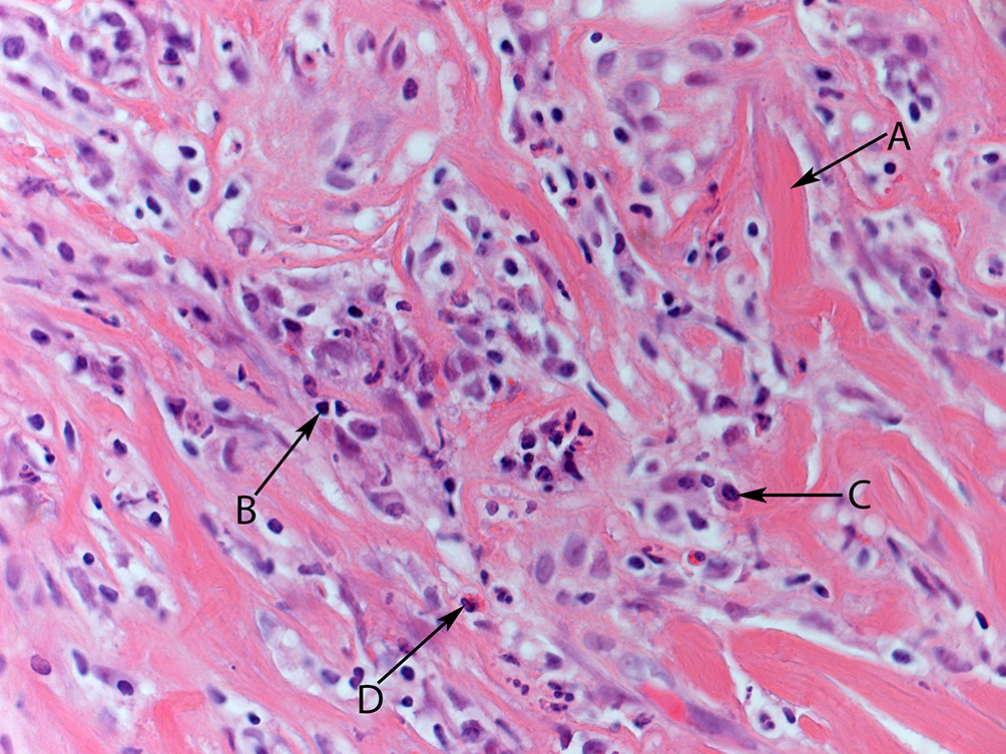
Coarse collagenous fibrous bundles with chronic inflammatory cells. A: collagenous bundles; B: lymphocytes; C: plasma cells; D: eosinophils. (H&E ×40).

The histopathological specimens, from both the right axillary and left thyroid biopsies (Figures 6 and 7), showed dense collagenous fibrous tissue with chronic inflammation cells, including lymphoplasmacytic infiltrations with scattered eosinophils. Due to non-concordance of clinical findings and imaging with histological findings, the case was discussed at a tertiary centre, where the images and histological specimens were reviewed. There was no evidence of malignancy at either site, with the morphology and immunophenotype being in favour of IgG4-related sclerosing fibroinflammatory disease.

Subsequently, the patient’s serum IgG4 level was checked. This was not elevated at 1.08 g l^−1^ (normal range <1.35 g l^−1^) with normal complement levels and IgG being just above the upper limit of normal at 16.2.

Following multidisciplinary team discussion and a diagnosis of IgG4-mediated sclerosing fibroinflammatory disease, the patient was commenced on oral prednisolone 40 mg daily for a month to be tapered in accordance with response. She stayed on this for 3 months until Mycophenolate 1g twice a day was introduced as a steroid sparing agent. The patient showed good clinical and imaging response and the steroids were weaned off over the course of 2 months. She subsequently developed primary hypothyroidism for which she was started on Levothyroxine 50 mcg.

## Discussion

IgG4-mediated sclerosing fibro-inflammatory disease is a rare systemic disease which has the ability to form inflammatory masses and to mimic tumours, with almost every organ having the potential to be affected. Although it is a rare entity, it should be considered particularly when a patient presents with two or more separate organs involved concurrently in a disease process. The appearances can readily be interpreted as malignant disease. It is essential to recognise and diagnose this condition as early as possible in order to commence treatment to avoid permanent organ damage. The disease can masquerade as a malignancy and is an important differential when suspecting a primary malignancy in two separate organs.^1^

The pathophysiology of this condition is still not understood, but it is thought to be autoimmune in nature. Affected tissues are characterised by lymphoplasmacytic infiltrate which is rich in IgG4-positive plasma cells. This may lead to chronic inflammation, fibrosis and phlebitis. These changes are the foundation for pathological diagnosis of this disease.^2,3^

It is more common in middle aged and older males, but when affecting the head and neck region males and females appear to be affected equally. Around 40–80% of patients with IgG4-related disease have regional lymphadenopathy and a history of asthma or other allergy is not uncommon.^4,5^

The clinical presentation of IgG4-related sclerosing fibroinflammatory disease varies depending on the organ involved. Ultimately any organ can be affected.^4^ It may be localised to one or two organs, or can present with diffuse multiorgan disease with swelling of the organ involved, known as an inflammatory pseudotumour. A relatively common presentation is autoimmune pancreatitis.^6,7^ Other gastrointestinal manifestations include idiopathic retroperitoneal fibrosis (Ormond’s disease^8^), gastritis, hepatitis^9^ and sclerosing cholangitis.^10^ Thyroid involvement may be associated with hypothyroidism with Riedel’s thyroiditis.^11^ Eye disease may present as an inflammatory pseudotumour of the orbit.^12^ Heart involvement may present as sclerosing aortitis, periaortitis and constrictive pericarditis.^13^ Lung, kidney and skin disease may present as inflammatory pseudotumours with glomerulonephritis and interstitial pneumonitis also possible presentations. Hypertrophic pachymeningitis is also a rare presentation of the disease^14^ as well as prostatitis and sialadenitis.^15^

This case in particular consisted of an unusual manifestation of the disease as it involved both the thyroid and the axilla simultaneously, but also gave secondary oedema changes in the breast mimicking inflammatory breast cancer, not previously described to our knowledge.

Inflammatory breast cancer is an uncommon but aggressive disease in which lymph vessels in the skin of the breast become blocked by the malignant cells.^16^ It is termed inflammatory as the breast usually looks erythematous, swollen and inflamed. There are a number of conditions which can mimic inflammatory breast malignancy, including infection, fat necrosis, granular cell tumours, spindle cell lesions, fibromatosis, myofibroblastoma, Mondor's disease, nodal mass and granulomatous mastitis among others.

Diagnosis of IgG4-related disease may prove difficult due to the potential to involve multiple organs simultaneously and its potential to mimic malignant disease. There are no definitive diagnostic criteria, but key factors in diagnosing suspected cases involve a combination of imaging findings, specific biopsy histological findings and ultimately a sensitivity to corticosteroids.^17^

Serology may show a peripheral eosinophilia and IgG4 is raised in many patients. Some patients however, may present with normal IgG4 levels (even prior to treatment). Serum IgG4 cannot be used alone for diagnosis as its levels are neither satisfactorily sensitive nor specific for this disease. Tissue diagnosis consists of variable degree of storiform fibrosis (scarring which entails cells arranged in a cartwheel like manner), and lymphoplasmacytic infiltration.^17^

Glucocorticoids are the mainstay of treatment usually starting with high dose prednisolone 40 mg a day for about 2 to 4 weeks with gradual weaning down. For people who cannot be taken off steroids, steroid sparing agents such as Azathioprine, Rituximab, methotrexate and mycophenolate are utilised. Mycophenolate has been used in one study with significant improvement when used in combination with Prednisolone.^18–20^

Treatment may also include management of organ specific issues *e.g*. thyroxine in thyroid-related disease, insulin in diabetes mellitus, pancreatic enzyme replacement in pancreatic insufficiency.

Multiple organs may continue to become involved gradually despite what appears to be effective treatment. Significant benefit may be seen from treatment; however, a large proportion of patients relapse on discontinuation. Disease prognosis is variable and it may even resolve unexpectedly or persist with a relapsing and remitting course. In untreated patients, indicators of poor prognosis include cirrhosis, portal hypertension, biliary obstruction, retroperitoneal fibrosis, complications from aortic involvement (dissections in particular), diabetes mellitus and pancreatic failure.

A history of malignancy has been suggested as a risk factor for this condition. Its association with possible development of malignant disease however, is controversial. Some studies have shown an increased risk of developing malignancy with IgG4-related disease, in particular non-Hodgkins lymphoma; however, other studies have refuted this.^21^

Overall, IgG4-mediated inflammatory disease is a rare but increasingly recognised disease, with a wide spectrum of clinical presentations and the ability to mimic malignancy. It should be considered in the differential diagnosis of tumour-like lesions in multiple organs, and early biopsy diagnosis is essential.

## Learning points

1. IgG4-mediated sclerosing fibro-inflammatory disease is a rare systemic disease and presentation may vary.
2. It may be localised to one or two organs, or can present with diffuse multiorgan disease.
3. It has the ability to mimic malignancy.
4. Early biopsy diagnosis is essential in order to facilitate early intervention and treatment.
5. It is responsive to steroids which are the mainstay of treatment.
